# Optimizing Image Segmentation for Microstructure Analysis of High-Strength Steel: Histogram-Based Recognition of Martensite and Bainite

**DOI:** 10.3390/ma19020429

**Published:** 2026-01-22

**Authors:** Filip Hallo, Tomasz Jażdżewski, Piotr Bała, Grzegorz Korpała, Krzysztof Regulski

**Affiliations:** 1 Faculty of Metals Engineering and Industrial Computer Science, AGH University of Krakow, al. A. Mickiewicza 30, 30-059 Kraków, Poland; hallo@agh.edu.pl (F.H.); pbala@agh.edu.pl (P.B.); regulski@agh.edu.pl (K.R.); 2 Institute of Metal Forming, TU Bergakademie Freiberg, 09599 Freiberg, Germany

**Keywords:** machine learning, neural networks, microstructure, bainite, martensite, high strength steel, slic, superpixel, segmentation, bayesian optimization

## Abstract

This study systematically compares three unsupervised segmentation algorithms (Simple Linear Iterative Clustering (SLIC), Felzenszwalb’s graph-based method, and the Watershed algorithm) in combination with two classification approaches: Random Forest using histogram-based features and Convolutional Neural Networks (CNNs). The study employs Bayesian optimization to jointly tune segmentation parameters and model hyperparameters, investigating how segmentation quality impacts downstream classification performance. The methodology is validated using light optical microscopy images of a high-strength steel sample, with performance evaluated through stratified cross-validation and independent test sets. The findings demonstrate the critical importance of segmentation algorithm selection and provide insights into the trade-offs between feature-engineered and end-to-end learning approaches for microstructure analysis.

## 1. Introduction

Accurate characterization of steel microstructures is essential for understanding material properties and optimizing manufacturing processes. However, manually segmenting steel micrographs remains highly challenging due to the inherent complexity of metallographic images [1]. Steel surfaces often contain irregularities such as scratches, corrosion products, stains, and heterogeneous textures, all of which complicate the identification of distinct structural boundaries. Moreover, the reflective nature of steel introduces glare, reflections, and shadowing effects that can obscure fine details, particularly in light microscopy (LM) images. As a result, manual segmentation is often time-consuming, subjective, and requires considerable metallographic expertise [2].

Among the various steel microstructural phases, bainite presents particularly subtle and complex morphological characteristics that make its identification especially challenging [3]. Bainitic structures exhibit fine-scale features and internal morphological variability that can be difficult to differentiate [4]. Accurate characterization often depends not only on visual appearance, but also on contextual knowledge such as alloy composition, thermomechanical history, and transformation kinetics, which experts use to guide morphological interpretation [5]. Differentiating bainite from morphologically similar phases (most notably martensite) is particularly problematic in LM images, where both can appear nearly indistinguishable because of limited resolution and optical artifacts.

A wide range of methods have been proposed for the classification of microstructural phases in steels [6,7]. These include approaches based on cementite morphology, local substructure density, regional contour patterns, or entropy-based descriptors used to segment ferrite, bainite, and martensite. More recently, convolutional neural networks (CNNs) such as U-Nets have been applied to treat microstructure analysis as an image segmentation problem, leveraging their strong feature-extraction capabilities in complex metallographic images [8]. Existing classification frameworks also differ in their treatment of bainitic structures: the widely adopted upper/lower bainite distinction contrasts with more comprehensive systems such as that of Zajac [9], which identify multiple bainitic morphologies, including granular bainite composed of irregular ferrite and diverse carbon-enriched constituents. Such frameworks highlight the structural diversity within bainite and underscore the difficulty of developing automated classification tools [10].

The characterization of bainite and martensite has been mainly based on Scanning Electron Microscopy (SEM), which provides high-resolution imaging capable of revealing fine substructures [11]. However, SEM requires extensive sample preparation, is costly to operate, and requires specialized expertise. In contrast, LM remains the predominant imaging modality in industrial environments due to its accessibility, low cost, and rapid sample throughput. Despite these advantages, LM lacks the resolving power needed to unambiguously capture fine bainitic features, making automated or manual classification a highly challenging task [2]. This limitation underscores the need for robust machine learning (ML) approaches capable of extracting discriminative information from real-world LM images.

Previous ML studies on histogram-based LM micrograph analysis have focused primarily on the classification of bainitic structures [12], but have not addressed the critical problem of distinguishing bainite from martensite, nor have they systematically examined how different unsupervised segmentation algorithms influence downstream classification performance. Furthermore, little attention has been given to the joint optimization of segmentation parameters and model hyperparameters. Although robust software tools [13] and established AI frameworks exist for general microstructure segmentation, the specific challenge of bainitic steel classification presents unique difficulties that extend beyond the application of standard neural architectures. The high morphological similarity between bainite and martensite in ferrous metallurgy introduces significant ambiguity in automated classification, particularly in LM images where fine substructural differences are not readily discernible. This morphological overlap, combined with the inherent variability of bainitic structures, creates a classification problem that requires careful methodological consideration of both segmentation quality and feature representation to effectively bridge classical metallographic analysis with automated AI-driven phase identification for these morphologically similar and difficult-to-classify microstructural constituents.

This article addresses these research gaps by developing and evaluating a machine learning framework to detect and classify martensite and bainite in high-strength steel microstructures using LM images. The study systematically compares three unsupervised segmentation algorithms: Simple Linear Iterative Clustering, Felzenszwalb’s graph-based method and the Watershed algorithm—combined with two classification strategies: Random Forest models based on histograms and Convolutional Neural Networks. Bayesian optimization is used to jointly tune segmentation parameters and classifier hyperparameters, enabling an integrated assessment of how segmentation quality affects classification accuracy. The methodology is validated using LM micrographs of a high-strength steel sample, with performance evaluated through stratified cross-validation and independent test sets. The results highlight the critical importance of segmentation algorithm selection and provide insights into the trade-offs between feature-engineered and end-to-end learning approaches for microstructure classification.

## 2. Material and Methods

Research was conducted on a steel sample subjected to a semi-continuous rolling process. The chemical composition of the steel is presented in Table 1.

The sample, which has the shape of a 2 cm long wire, was mounted in a dilatometer where heat treatment was performed and phase transformation was observed through dilatometric measurements.

The specimen was subjected to a controlled thermal process consisting of three main stages. First, the sample was heated to a temperature of 1000 °C. After reaching this temperature, it was maintained for 3 min to ensure uniform thermal exposure. Subsequently, the specimen was rapidly cooled to 50 °C with a cooling speed of 70 m/s.

The heating process was carried out under vacuum conditions, with an initial pressure of $1.0\times 10^{-1}\;\mathrm{mbar}$. The cooling was carried out using nitrogen gas (N_2_) at a pressure of 6bar, to ensure a controlled and accelerated temperature decrease.

**Figure 1:**
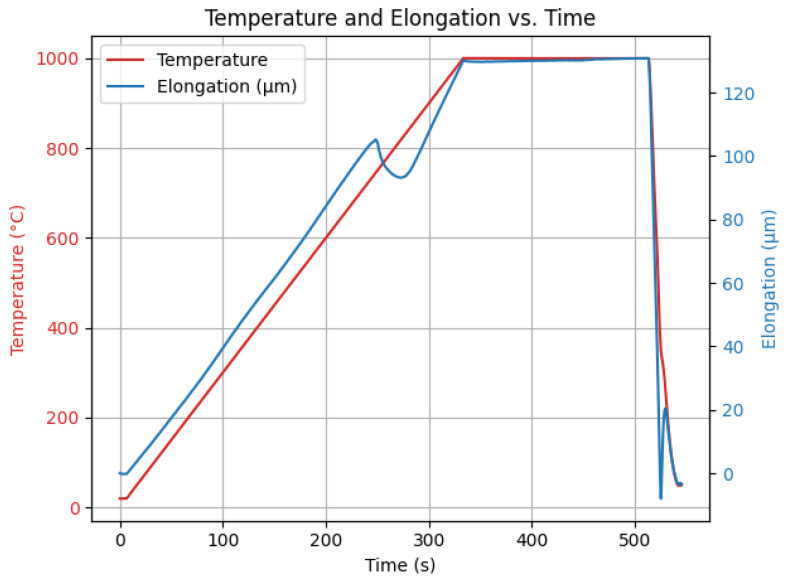
Dilatometric results for the sample.

Throughout the experiment, all relevant parameters were monitored and recorded in real time by the measurement system. The setup continuously generated and displayed graphical data that represent the evolution of the process variables. The results of the experiment are presented in Figure 1. The final seconds of the process (after approximately 500 s) may appear more chaotic because of the dynamics of the system, which in turn leads to higher measurement uncertainty of the instrumentation.

After undergoing heat treatment, the specimen was sectioned along its longitudinal axis and embedded in black bakelite resin containing a carbon filler using a hot-mounting process. The mounted sample was then subjected to a sequential polishing procedure, progressing through increasingly fine abrasive grades, followed by final polishing and chemical etching. Etching was performed using a 3% nitric acid (HNO_3_) solution in ethanol, with the sample immersed for about 8 s. Finally, the samples were cleaned with pure ethanol.

### 2.1. Microstructure Imaging

**Figure 2:**
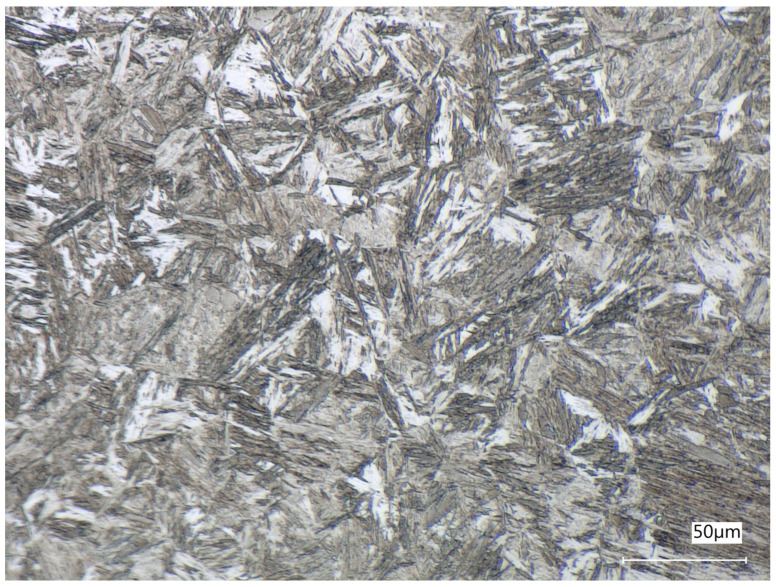
Example of a sample micrograph with bainitic and martensitic regions.

A total of six micrographs of the sample were taken. Consistent imaging parameters were applied, ensuring uniform lighting and a fixed magnification of 1000× across all images. A sample result is presented in Figure 2.

**Figure 3:**
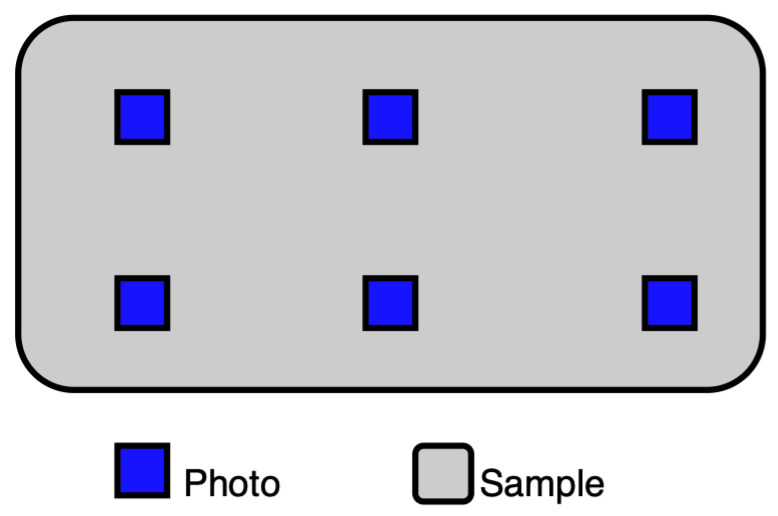
Sample section with spots where images were taken.

To ensure that local anomalies do not affect future analyses, photos were taken in 6 different places, as presented in Figure 3.

### 2.2. Image Annotation

Images of the steel sample were annotated by an expert in metallurgy using labelme 5.8.1, as a package for Python 3.10 that provides a graphical user interface. Two classes of microstructure were distinguished: martensite and bainite. These regions were annotated using polygons. The labeling software saves output as a JSON file with entries named after the microstructure type containing coordinates of the polygon points. Subsequently, segmentation of the annotated regions was employed. These regions were extracted using custom Python code; each region was processed on the fly as a single image that was segmented by unsupervised segmentation algorithms.

### 2.3. Segmentation Algorithms

To increase the number of segments generated by the model, unsupervised segmentation was applied to the annotated regions. This approach not only led to a substantial increase in the total segment count but also advanced the image processing pipeline toward a more automated form of microstructure analysis. As a result, smaller, well-defined segments could be extracted from the images without human intervention. In this process, three segmentation algorithms were used: Simple Linear Iterative Clustering, Felzenszwalb’s algorithm and the Watershed method.

Simple Linear Iterative Clustering (SLIC) is a superpixel-based segmentation algorithm that groups pixels according to both color similarity and spatial proximity. The method operates in a five-dimensional space defined by the CIELAB [14] color components and the image coordinates, ensuring that the resulting superpixels are compact and uniform. SLIC’s efficiency and adherence to object boundaries make it particularly suitable for applications requiring detailed region-based analysis or feature extraction [15].

Felzenszwalb’s algorithm adopts a graph-based representation of the image, where each pixel corresponds to a node and edges connect adjacent pixels. The segmentation process relies on minimizing internal differences within regions while preserving discontinuities between them [16]. This approach allows the algorithm to produce variable segment sizes depending on local image characteristics, providing a balance between oversegmentation and undersegmentation. Its efficiency and sensitivity to structural variations make it effective for complex natural images.

The Watershed method conceptualizes the grayscale image as a topographical surface, where pixel intensities represent elevation values. By simulating a flooding process from regional minima, the algorithm delineates boundaries along watershed lines that separate distinct catchment basins [17]. While the method can be prone to oversegmentation due to image noise and minor intensity variations, this limitation can be mitigated through pre-processing techniques such as Gaussian smoothing or gradient-based filtering.

### 2.4. Classification Algorithms

#### 2.4.1. Random Forest (RF)

The Random Forest algorithm [18] has gained considerable attention due to its robustness, interpretability, and strong predictive performance, as demonstrated in our previous experiments. Random Forest is an ensemble-based method that builds a collection of decision trees, where each tree is trained on a random subset of the data and features. The final prediction is determined by aggregating the outputs of all trees, typically using majority voting in classification or averaging in regression tasks.

The fundamental idea behind Random Forest lies in the principle of “wisdom of the crowd”—combining the predictions of many weak learners (decision trees) to obtain a more accurate and stable model. Each tree in the ensemble contributes to reducing variance without significantly increasing bias, which helps to overcome the overfitting problem often observed in single decision trees.

Random Forest offers several advantages compared to other classification algorithms such as logistic regression, support vector machines (SVMs), or k-nearest neighbors (KNN). It is less sensitive to noise and outliers, can naturally handle missing values, and does not require extensive data preprocessing or feature scaling. Moreover, it can efficiently model nonlinear relationships and complex feature interactions that linear models often fail to capture. Another notable benefit is its ability to estimate feature importance, allowing researchers to identify which variables contribute most significantly to the prediction task.

Due to its balance between interpretability, flexibility, and predictive power, Random Forest is widely used across disciplines such as bioinformatics, finance, environmental modeling, and engineering. In this work, it was chosen as the core classification algorithm because of its proven ability to deliver high accuracy with relatively low risk of overfitting and minimal parameter tuning.

#### 2.4.2. Convolutional Neural Networks

Convolutional Neural Networks represent one of the most powerful and widely used architectures in modern machine learning [19], particularly for image, signal, and spatial data analysis. Unlike traditional fully connected neural networks, CNNs are specifically designed to exploit the spatial structure of data by applying convolutional filters that detect local patterns such as edges, textures, and shapes. These local features are then combined hierarchically to form higher-level abstractions, allowing CNNs to automatically learn relevant representations directly from raw data.

The basic building block of a CNN is the convolutional layer, which performs a series of convolutions between the input data and a set of learnable filters (kernels). Each filter extracts a specific type of feature map, emphasizing certain spatial patterns. Pooling layers are subsequently used to reduce dimensionality and computational complexity by summarizing feature map information, making the network more invariant to small translations and distortions. Finally, fully connected layers integrate these extracted features for classification or regression tasks, depending on the application.

Compared to traditional machine learning algorithms such as Random Forest, Support Vector Machines, or k-Nearest Neighbors, CNNs offer a major advantage: they eliminate the need for manual feature engineering. Instead of relying on hand-crafted descriptors, CNNs automatically discover features that are optimal for the target task. This makes them especially effective in fields like computer vision, remote sensing, and medical imaging, where feature extraction from high-dimensional data is complex and domain-specific.

CNNs also demonstrate remarkable scalability and adaptability. With sufficient data and computational resources, they can learn intricate and nonlinear relationships that simpler models cannot capture. Moreover, transfer learning allows pre-trained CNNs to be fine-tuned for new tasks with relatively small datasets, significantly reducing training time and improving generalization.

However, CNNs are not without limitations. They typically require large amounts of labeled data, significant computational power, and careful architecture design to avoid overfitting. Despite these challenges, their unparalleled ability to extract hierarchical representations from raw data has made them the state-of-the-art choice in many pattern recognition and classification problems.

In this study, a Convolutional Neural Network was implemented to classify input data by learning spatial dependencies through successive convolutional and pooling operations. The architecture was optimized using hyperparameter tuning techniques to ensure high performance, robustness, and efficient generalization across validation datasets.

### 2.5. Hyperparameter Tuning Algorithms

In this study, Bayesian Optimization was used as a hyperparameter tuning algorithm, as it is a probabilistic, model-based approach that efficiently explores the hyperparameter space [20]. Optuna was the library used as an implementation of this algorithm [21].

Bayesian Optimization works by building a surrogate model, often a Gaussian Process (GP), that approximates the relationship between hyperparameter configurations and model performance. This surrogate model is iteratively updated as new evaluations are performed. An acquisition function, such as Expected Improvement (EI) or Upper Confidence Bound (UCB), is then used to determine the next set of hyperparameters to evaluate. This mechanism allows the algorithm to balance exploration of uncertain regions with exploitation of known promising areas in the search space.

Compared to traditional grid search or random search, Bayesian Optimization offers significant advantages. It requires fewer model evaluations to find optimal or near-optimal parameters, which is particularly beneficial when model training is computationally expensive. Furthermore, it can capture complex, nonconvex relationships between hyperparameters and performance metrics, where simpler methods may fail.

Another key advantage is its adaptability—Bayesian Optimization does not assume any specific structure of the objective function and can be applied to a wide range of algorithms, including Random Forest, gradient boosting, and deep learning models. This makes it a versatile and efficient tool for automatic model tuning. In this study, Bayesian Optimization was applied to optimize key Random Forest parameters, enhancing classification accuracy while minimizing computational overhead.

### 2.6. Software Development

**Figure 4:**
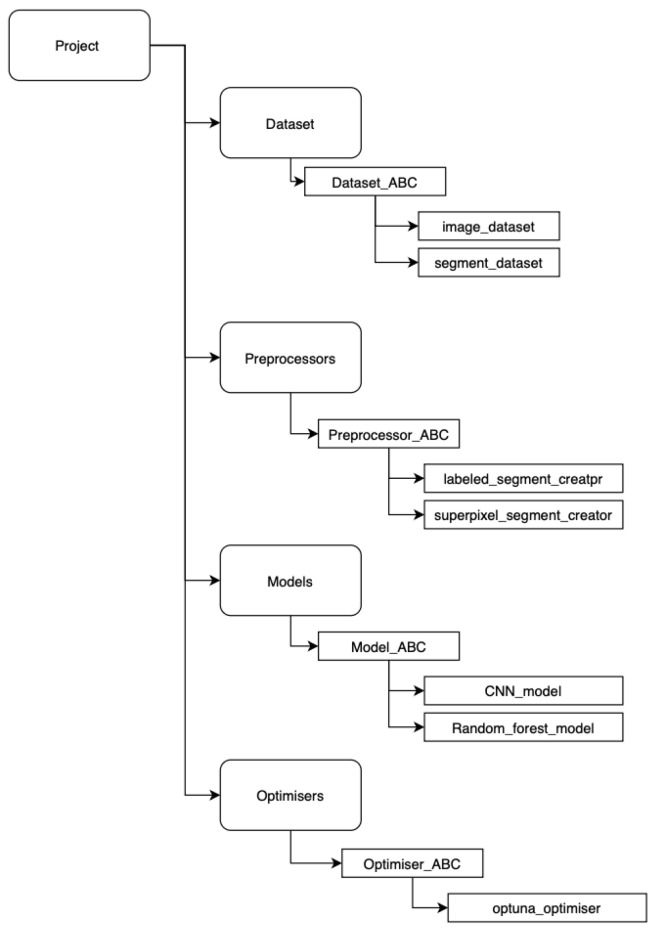
Code modules.

The data tracking system was developed using the MLflow 3.1.1 framework [22]. Each stage of the data preparation pipeline—including segmentation, post-processing, and related tasks—was implemented as an independent module. Within each module, an abstract base class (ABC) was defined to facilitate the integration of new algorithms without impacting existing implementations (see Figure 4). This modular design enables the execution of new experiments using the same input data, ensuring reproducibility and extensibility of the workflow.

A self-service interface was implemented in Python 3.10 using Jupyter Notebook, allowing users to utilize predefined modules and visualize intermediate and final results interactively.

In addition, to ensure that all components can communicate with each other and to reduce the number of errors, all contracts have been implemented in a dataschema class. This allows modules to be reused for future work.

## 3. Classification Pipeline

The processes of unsupervised segmentation, histogram construction for the Random Forest Classifier or direct passage of the segments to the Convolutional Neural Networks, and classification were orchestrated under the control of the Bayesian optimizer.

To ensure robust evaluation, the training process employed stratified 5-fold cross-validation, in which the dataset was partitioned into five folds preserving class proportions across each split. In every iteration, four folds (80% of the data) were used for training and one fold (20%) for testing. The classification performance was optimized by maximizing the average F1-score computed across all five folds, ensuring that both precision and recall were balanced during model selection.

### 3.1. Segmentation Parameter Optimization

**Figure 5:**
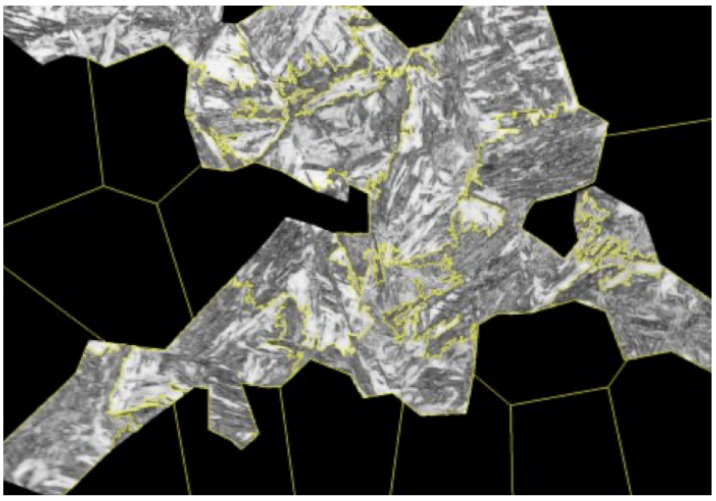
SLIC segment sample.

The parameters of the image segmentation algorithms (sample presented in Figure 5) were tuned to enhance the quality and consistency of the extracted regions. For the SLIC algorithm, the varying size of the annotated regions rendered the direct optimization of the number of segments (n_segments) impractical. As this parameter would remain fixed across all annotated regions, optimizing it without accounting for differences in region size would likely result in suboptimal segmentation performance. To mitigate this effect, a new parameter—the number of pixels per superpixel (pixels_per_superpixel)—was created along with a mechanism recalculating n_segments’ individually for each annotated region (see Equation (1), Algorithm 1).

(1) $$n_{i}=\left\lceil \frac{A_{i}}{p}\right\rceil ,$$

where $A_{i}$ denotes the total number of pixels in the region $R_{i}$, and *p* is the desired number of pixels per superpixel. The ceiling function ⌈·⌉ ensures that $n_{i}$ is always rounded to the nearest integer, ensuring that each region has enough superpixels to cover all its pixels. This maintains a consistent superpixel density across regions of varying sizes.
**Algorithm 1:** Adaptive SLIC Segmentation Based on Region Size.
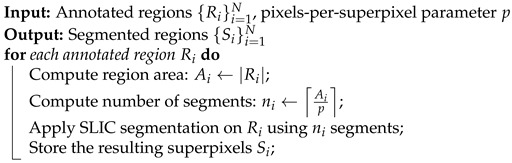


### 3.2. Histograms with Random Forest Classifier

**Figure 6:**
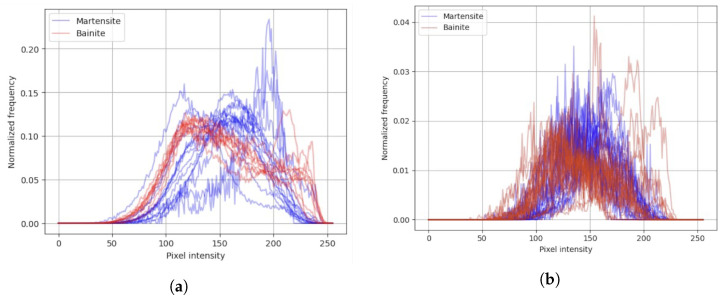
Comparative pipeline illustrating the differences in pixel intensity histograms derived from (**a**) expert manual classification and (**b**) the Felzenszwalb segmentation algorithm.

In this approach, the input information is a histogram (example presented in Figure 6) based on output segments from unsupervised algorithms.

#### Model Training and Optimization

**Figure 7:**
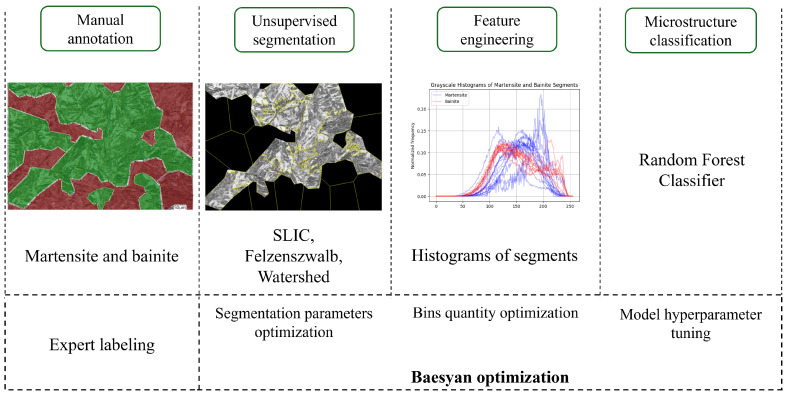
Pipeline of the image processing and segment classification using Random Forest Classifier.

At the initial stage of the workflow, a Random Forest model was employed to perform classification tasks. The overall schema of the implemented pipeline is illustrated in Figure 7. To ensure optimal model performance, a systematic hyperparameter tuning procedure was conducted over 100 iterations of the optimizer. The set of tunable parameters included the number of trees in the ensemble (n_estimators, 50–200), the maximum tree depth (max_depth, 5–20), the minimum number of samples required to split an internal node (min_samples_split, 2–10), and the minimum number of samples required to be present at a leaf node (min_samples_leaf, 1–5). Additionally, the number of features considered when searching for the best split (max_features, 1–5) and the number of discretization bins used during feature preprocessing (bins, 64–256) were also optimized.

As an outcome, parameters for the optimization included the number of pixels per superpixel (pixels_per_superpixel, 8000–30,000), compactness (compactness, 0.4–1.0), and the Gaussian smoothing factor (sigma, 0.1–1.5).

For the Felzenszwalb segmentation algorithm, the optimized parameters were the scale of observation (scale, 2–200), the minimum component size (min_size, 10–300), and the Gaussian smoothing parameter (sigma, 0.1–1.5). In a refined configuration, these ranges were adjusted to (scale, 160–350), (min_size, 250–500), and (sigma, 0.3–0.6), respectively, to improve segmentation granularity and boundary accuracy. All parameters were systematically explored to identify configurations that achieved the best trade-off between segmentation precision and the subsequent model interpretability. In contrast to SLIC, the parameter controlling the segment scale was independent of the annotated region size. Consequently, during the optimization process, this parameter could be directly adjusted by the optimizer.

A summary of the pipeline for this approach is presented in Figure 7.

### 3.3. Convolutional Neural Network Application

**Figure 8:**
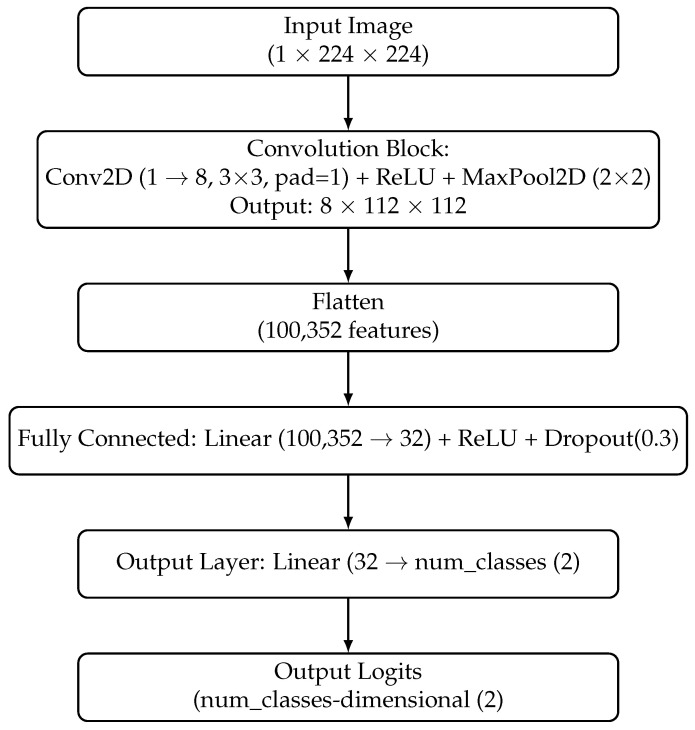
Architecture of the Custom 1-Layer Grayscale CNN.

**Figure 9:**
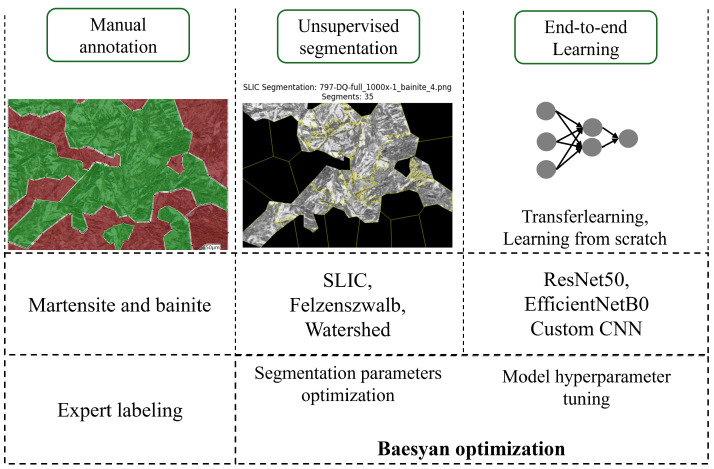
Pipeline of the image processing and segment classification using Convolutional Neural Networks.

During the second stage of the experiment, Convolutional Neural Networks were utilized to classify segments obtained by SLIC segmentation. Three types of CNNs were applied: ResNet50, EfficientNetB0, and a Custom Network with one convolutional layer (see Figure 8), which was trained from scratch without transfer learning. The pipeline is presented on Figure 9.

Due to the variable size of segments and fixed size of the inputs to the CNN model, all segments were scaled using the cv2.INTER_AREA function to a size of 224×224, which is typical for many CNNs, including ResNet50 and EfficientNetB0. The segmentation parameter and model hyperparameter search space was narrowed down: number of pixels per superpixel (pixels_per_superpixel, 14,000–20,000), compactness (compactness, 0.65–0.75), and the Gaussian smoothing factor (sigma, 1–1.4). The model hyperparameter search space was set by learning rate (lr, $2\times 10^{-2}$–$8\times 10^{-2}$) and the number of epochs (epochs, 1–60). After 50 iterations of the optimizer, the search space was narrowed down to lr, $2\times 10^{-2}$–$8\times 10^{-2}$ and epochs, 20–60.

## 4. Results

### 4.1. Validation Results for Histogram Classification Using RF

**Figure 10:**
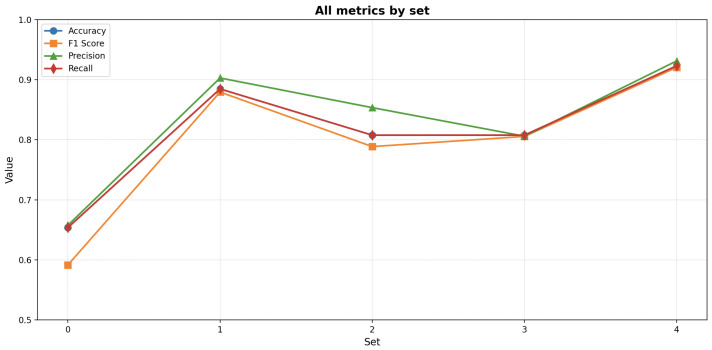
All metrics by set for a SLIC algorithm.

After the optimization process, results were analyzed to reveal the relationships between segmentation parameters, RF hyperparameters, and the corresponding averaged classification metrics for all folds; the values per fold are presented on Figure 10.

The SLIC segmentation demonstrated a well-defined cluster of high-performing solutions, with F1-scores reaching 0.88. The most effective models were typically obtained with moderate values of n_estimators (approximately 150–200) and max_depth (15–20), combined with balanced segmentation settings such as intermediate values of pixels_per_superpixel (10,000–16,000) and sigma (0.2–0.4). Furthermore, higher bins (200–250), min_samples_split (6–8), and min_samples_leaf values (3–4) appeared to enhance model metrics. The performance metrics (accuracy, precision, recall, and F1) were strongly correlated, suggesting balanced classification performance across all evaluation criteria.

The Felzenszwalb segmentation achieved slightly lower overall results, with F1-scores reaching 0.86. High-performing cases were concentrated within a narrow region characterized by moderate scale values (approximately 300–450), min_size within the range (1100–1600), sigma levels within the range (0.3–0.5), and bins within the range (66–150), with higher n_estimators (150–200). However, the relationships between segmentation parameters and classification performance were less distinct, indicating higher sensitivity of the model to hyperparameter variations.

The Watershed segmentation achieved overall the lowest results, with F1-scores reaching 0.56. Analysis of model hyperparameters did not indicate a cluster of high-performing cases. However, analysis of segmentation parameters revealed that the highest results were observed when connectivity values reached 4 and bins were within the range 150–200.

#### Validation Results for CNN

Due to the increased computational workload while training CNN models, only SLIC segmentation, which demonstrated the highest score in the previous step, was utilized to provide segments for the CNNs.

EfficientNetB0 trained with IMAGENET1K_V1 as initial weights achieved at most an F1-score of 0.79. The best models were trained with hyperparameters such as lr in the range (0.05–0.075) and 40–60 epochs. EfficientNetB0 trained from scratch achieved at most an F1-score of 0.65. The best models were trained with hyperparameters such as lr in the range (0.03–0.08) and 50–60 epochs.

ResNet50 trained with IMAGENET1K_V2 as initial weights achieved at most an F1-score of 0.82. The best models were trained with hyperparameters such as lr in the range (0.045–0.055) and 54–60 epochs.

The custom CNN achieved at most an F1-score of 0.82. The best models were trained with hyperparameters such as lr in the range (0.03–0.08) and 45–60 epochs.

### 4.2. Evaluation on the Independent Test Dataset

Following the hyperparameter optimization phase, the generalization capability of the proposed models was evaluated using an independent test dataset. As detailed in Section 2, this dataset comprises three micrographs derived from the same sample material. Ground truth annotations were provided by a domain expert, identifying a total of 20 bainitic and 33 martensitic regions.

The Random Forest (RF) classifier was deployed using the optimal configuration identified during validation. Specifically, the histogram feature extraction was performed with 241 bins. The SLIC segmentation algorithm was parameterized with a target region size (pixels_per_superpixel) of 11,843, a compactness factor of 0.79, and a sigma smoothing value of 0.62. The quantitative performance of the RF model is detailed in Table 2 and Table 3.

Subsequently, the Convolutional Neural Network (CNN) was evaluated. The network was trained for 60 epochs with a learning rate of 0.045 based on Adam Optimiser with batches equal to 32. For this approach, the optimal SLIC segmentation parameters differed slightly from the RF configuration, utilizing a superpixel size of 15,635, compactness of 0.66, and sigma of 1.0. The classification results yielded by the CNN model are presented in Table 4 and Table 5.

## 5. Discussion

Overall, the comparison of the experimental setups indicates that the SLIC and Felzenszwalb configurations provided more robust and generalizable results. The Watershed configuration exhibited reduced consistency and lower performance.

The superior performance of SLIC segmentation (F1-scores reaching 0.88) can be attributed to its ability to generate compact, uniform superpixels that effectively capture local textural and intensity characteristics of bainitic and martensitic microstructures. The algorithm’s operation in the five-dimensional CIELAB color space, combined with spatial proximity constraints, ensures that resulting segments align well with microstructural boundaries while maintaining consistent size and shape properties. This consistency is particularly advantageous for histogram-based feature extraction, providing stable representations of local intensity distributions. The well-defined cluster of high-performing solutions observed during optimization suggests that SLIC parameters can be tuned effectively within a relatively broad range, with optimal configurations identified at intermediate values of pixels_per_superpixel (10,000–16,000) and moderate sigma (0.2–0.4).

Felzenszwalb’s algorithm demonstrated comparable performance (F1-scores reaching 0.86), though with slightly lower overall metrics and higher sensitivity to hyperparameter variations. The graph-based approach proved effective for capturing variable-scale features present in steel microstructures, but the narrower parameter range yielding high performance (scale values 300–450, min_size 1100–1600) indicates that this method requires more precise tuning compared to SLIC. The algorithm’s ability to produce variable segment sizes can be advantageous for capturing both fine bainitic structures and larger martensitic regions, though this variability may introduce inconsistency in feature extraction when combined with histogram-based classification.

In contrast, Watershed segmentation exhibited significantly inferior performance (F1-scores reaching only 0.56) due to its inherent susceptibility to oversegmentation in the presence of noise and minor intensity variations typical in LM micrographs. The method’s sensitivity to artifacts such as scratches, stains, and uneven illumination leads to excessive fragmentation, producing segments that do not align well with actual microstructural boundaries. This oversegmentation results in histogram features that lack discriminative power, as segments capture noise and artifacts rather than meaningful microstructural characteristics.

The comparison between Random Forest and Convolutional Neural Network approaches reveals important trade-offs. The RF-based pipeline achieved superior generalization on the test dataset (accuracy 0.64, F1-score 0.63) compared to the CNN approach (ResNet50 accuracy 0.28, F1-score 0.24). This performance gap can be attributed to the compact, interpretable nature of histogram features that effectively capture intensity distribution characteristics distinguishing bainite from martensite and the inherent invariance of histogram features to spatial transformations, edge shapes, and minor segment size variations.

The CNN approach, despite achieving competitive validation performance (F1-scores up to 0.82), demonstrated poor generalization on the test set (additional feature exploration directly for the CNN problem may help in future research), with ResNet50 showing severe class imbalance issues (recall of 1.00 for bainite but only 0.03 for martensite). This discrepancy indicates overfitting, likely amplified by variations in segment-edge characteristics between the training and test datasets, the inherent sensitivity of the CNN model to edge-related features, the limited dataset size [23], the requirement to resize segments to a fixed 224 × 224 resolution—which may introduce artifacts or reduce spatial fidelity—and the intrinsic challenges associated with transferring pre-trained models developed for natural images to metallographic micrographs.

The test results reveal important limitations, with the RF model’s performance on the test set (accuracy 0.64) representing a significant drop from validation performance (F1-score 0.88). This performance degradation may arise from differences between the test region properties and those of the training set, such as variations in annotated region shape and boundary characteristics, which, after application of unsupervised segmentation with parameters optimized during the validation process, lead to segments that significantly diverge from those in the training dataset, despite the use of consistent processing parameters. Additional degradation may stem from variations in sample preparation, such as non-uniform etching, or from differences in imaging parameters across regions of the same sample. The asymmetric performance metrics (bainite F1-score 0.56 vs. martensite F1-score 0.70) suggest that bainitic structures may exhibit greater morphological variability not fully captured by histogram features.

These findings highlight the critical importance of segmentation quality in microstructure analysis pipelines. The substantial performance differences between segmentation methods demonstrate that the choice of segmentation algorithm is not merely a preprocessing step but a fundamental component that directly impacts downstream classification performance. Future research should focus on expanding datasets to include multiple samples with varying conditions, exploring additional feature extraction methods such as texture descriptors or domain-specific pre-trained models, investigating ensemble methods, addressing class imbalance issues, and integrating spatial context information to enhance discrimination between morphologically similar structures.

## 6. Conclusions

This study investigated the optimization of image segmentation algorithms for automated microstructure analysis of high-strength steel, focusing on differentiating between martensitic and bainitic structures using LM micrographs. Three unsupervised segmentation algorithms—SLIC, Felzenszwalb, and Watershed—were systematically evaluated in combination with two classification approaches: Random Forest using histogram features and Convolutional Neural Networks.

The key findings demonstrate that the choice of segmentation algorithm significantly impacts downstream classification performance. SLIC segmentation emerged as the most effective method, achieving F1-scores of up to 0.88 during validation, followed by Felzenszwalb’s algorithm (F1-scores reaching 0.86). Both methods demonstrated robustness and generated segments that align well with microstructural boundaries, allowing effective extraction of discriminative histogram features. In contrast, Watershed segmentation proved unsuitable, achieving only a 0.56 F1-score due to excessive oversegmentation caused by noise and artifacts typical in metallographic samples.

The comparison between Random Forest and CNN-based classification revealed important trade-offs. The histogram-based Random Forest approach demonstrated superior generalization on independent test data (accuracy 0.64, F1-score 0.63) compared to CNNs (accuracy 0.28, F1-score 0.24), despite achieving similar validation performance. This superior generalization can be attributed to the compact, interpretable nature of histogram features that effectively capture intensity distribution characteristics relevant for distinguishing bainite from martensite. CNN models, despite achieving competitive validation performance (F1-scores up to 0.82), showed poor generalization, indicating overfitting exacerbated by limited dataset size and challenges in transferring pre-trained models from natural images to metallographic micrographs.

Joint optimization of segmentation parameters and model hyperparameters using Bayesian optimization proved essential for achieving optimal performance. The adaptive SLIC segmentation approach, which adjusts segment count based on region size, demonstrated particular effectiveness by maintaining consistent superpixel density across regions of varying dimensions.

In conclusion, this research establishes that the combination of SLIC segmentation with histogram-based Random Forest classification provides a promising approach for automated bainite and martensite recognition in LM micrographs. The method offers a practical alternative to labor-intensive manual analysis while maintaining interpretability and computational efficiency. However, over a 20% performance degradation from validation to test sets, while 5-fold cross-validation was applied, indicates challenges in generalizing across different regions, highlighting the need for larger and more diverse datasets and addressing class imbalance issues. These findings emphasize that segmentation quality is not only a preprocessing concern but a fundamental component that directly determines the success of automated microstructure analysis pipelines.

## Figures and Tables

**Table 1:** Chemical composition of the analyzed steel.

C	Mn	Si	Ni	Cu	Mo	Nb + Ti + V
0.07	1.9	0.23	0.3	0.2	0.14	0.13

**Table 2:** Confusion Matrix for Bainite and Martensite Classification (RF algorithm).

	Predicted Bainite	Predicted Martensite
Actual Bainite	12	8
Actual Martensite	11	22

**Table 3:** Classification performance metrics using the Random Forest (RF) model.

Class	Precision	Recall	F1-Score	Support
Bainite	0.52	0.60	0.56	20
Martensite	0.73	0.67	0.70	33
Accuracy			0.64	53
Macro Avg	0.63	0.63	0.63	53
Weighted Avg	0.65	0.64	0.65	53

**Table 4:** Confusion Matrix for Bainite and Martensite Classification (CNN algorithm).

	Predicted Bainite	Predicted Martensite
Actual Bainite	10	0
Actual Martensite	28	1

**Table 5:** Classification performance metrics using the CNN model.

Class	Precision	Recall	F1-Score	Support
Bainite	0.26	1.00	0.42	10
Martensite	1.00	0.03	0.07	29
Accuracy			0.28	39
Macro Avg	0.63	0.52	0.24	39
Weighted Avg	0.81	0.28	0.16	39

## Data Availability

The original contributions presented in this study are included in the article. Further inquiries can be directed to the corresponding author.
